# A Risky Business? Habitat and Social Behavior Impact Skin and Gut Microbiomes in Caribbean Cleaning Gobies

**DOI:** 10.3389/fmicb.2019.00716

**Published:** 2019-04-09

**Authors:** Raquel Xavier, Renata Mazzei, Marcos Pérez-Losada, Daniela Rosado, Joana L. Santos, Ana Veríssimo, Marta C. Soares

**Affiliations:** ^1^CIBIO/InBIO, Centro de Investigação em Biodiversidade e Recursos Genéticos da Universidade do Porto, Porto, Portugal; ^2^Laboratoire d’Eco-Ethologie, Institut de Biologie, Université de Neuchâtel, Neuchâtel, Switzerland; ^3^Computational Biology Institute, Department of Epidemiology and Biostatistics, Milken Institute School of Public Health, The George Washington University, Ashburn, VA, United States

**Keywords:** *E. prochilos*, ecotypes, pathogens, coral-dwellers, sponge-dwellers, social contamination

## Abstract

The broadstripe cleaning goby *Elacatinus prochilos* has two alternative ecotypes: sponge-dwellers, which live in large groups and feed mainly upon nematode parasites; and coral-dwellers, that live in small groups or in solitude and behave as cleaners. Recent studies focusing on the skin and gut microbiomes of tropical fish showed that microbial communities are influenced mainly by diet and host species. Here, we compare the skin and gut microbiomes of the Caribbean broadstripe cleaning goby *E. prochilos* alternative ecotypes (cleaners and non-cleaners) from Barbados and predict that different habitat use and behavior (cleaning vs. non-cleaning) will translate in different bacterial profiles between the two ecotypes. We found significant differences in both alpha- and beta-diversity of skin and gut microbiomes belonging to different ecotypes. Importantly, the skin microbiome of obligate cleaners showed greater intra-sample diversity and harbored a significantly higher prevalence of potential fish pathogens. Likewise, potential pathogens were also more prevalent in the gut of obligate cleaners. We suggest that habitat use, diet, but also direct contact with potential diseased clientele during cleaning, could be the cause for these patterns.

## Introduction

Cleaner fish are pivotal players in marine ecosystems, providing a valuable service to other fish (known as clients) by removing ectoparasites and dead or damaged tissue (Côté, 2000). Classically described as “doctors of the sea,” these usually smaller-sized fish are conspicuously colorful organisms (Cheney et al., 2009) and are easily identified by client species. Clients then visit their territories (referred as cleaning stations) to be relieved of parasites, and to gain in stress reduction and other putative fitness benefits (Ros et al., 2011; Soares et al., 2011). Indeed, these cleaning interactions influence client growth rate (Clague et al., 2011; Waldie et al., 2011) and affect local populations mobility contributing to the diversity and abundance of juvenile and adult fish (Bshary, 2003; Grutter et al., 2003; Waldie et al., 2011). Additionally, the presence of cleaners is known to influence habitat selection (e.g., processes of settlement and recruitment) of young reef fishes (Sun et al., 2015a,b). Cleaners are primary agents in these mutualistic exchanges, choosing when and how to inspect clients, and determining the quality of these inspections. They can, for instance, select the number and size of the parasites they ingest (Grutter, 1997), decide whether to provide or not physical contact (tactile stimulation, Bshary and Würth, 2001) or the amount of bites given to fish mucus, which is harmful to the clients because mucus protects fish from disease and sunburn (Bshary and Grutter, 2002; Eckes et al., 2015). While there seems to be a great benefit of having such a decisive role, there is also vulnerability when one needs to wait for the food (clients) to visit. For instance, the amount of potential clientele could be determinant to the number of visits and dietary value may fluctuate, as parasite load of clients may change from reef to reef (Cheney and Côté, 2005), but also amongst client species (Soares et al., 2007) or between seasons (Grutter, 1994). Moreover, some clients could actually eat the cleaner (Côté, 2000), and cleaners are not indifferent to the risk involved (Soares et al., 2012). Finally, clients may also be vectors of parasites, bacterial contamination, and disease to cleaners (Grutter, 2002; Jones et al., 2004; Sasal et al., 2005). For example, cleaner fish (e.g., *Ctenolabrus rupestris* and *Centrolabrus exoletus*) used in salmon farms can become infected by pathogenic *Vibrio* sp. and *Aeromonas salmonicida*, as well as by pancreatic necrosis virus (Treasurer and Laidler, 1994), although little empirical evidence is available on this matter.

Bacterial consortia play an important role at all biological scales from individuals to ecosystems (McFall-Ngai et al., 2013; Gibbons and Gilbert, 2015; Delgado-Baquerizo et al., 2016). Fish microbiome studies have generally shown a high degree of host specificity and specialization across organs (e.g., Lowrey et al., 2015; Pratte et al., 2018; Rosado et al., 2019), as well as a close association between microbiome composition and host ecology. Specifically, host factors (ontogeny and genetic background), environment and diet are considered to be the main drivers of the bacteria consortia present in the fish gut (Tarnecki et al., 2017; Egerton et al., 2018) and skin (Larsen et al., 2013; Chiarello et al., 2018). Recent studies have also revealed crucial connections between animal microbiomes and social behavior; those studies have shown that social interactions and physical proximity can modulate the composition and function of animal microbiomes (White et al., 2010; Koch and Schmid-Hempel, 2011; Tung et al., 2015), and that the microbiome affects social communication by influencing the host central nervous system and peripheral chemical communication (Sharon et al., 2010; Theis et al., 2013; Venu et al., 2014). In fact, microbial transfer between socially interacting partners is beginning to be considered a key driver in the cost–benefit calculus of group network interactions (Koch and Schmid-Hempel, 2011; Tung et al., 2015). While microbe transmission may be beneficial by promoting disease resistance (Endt et al., 2010; Stecher et al., 2010; Koch and Schmid-Hempel, 2011), it may also facilitate pathogen transmission between interacting hosts (Garrett et al., 2010; Elinav et al., 2011). In this respect, cleaners could become good animal models to study the role of microbiome in mutualistic behavior, as both partners come in close physical contact during interactions, allowing for direct microbe transmission (skin-to-skin) and potentially also modulating the gut microbiome of cleaners (Soares et al., 2019).

In gobies, cleaner species occur exclusively in the genus *Elacatinus*, which include 27 described species that are distributed throughout the western Atlantic Ocean, from North Carolina (United States) to Brazil (Colin, 1975, 2010; Taylor and Hellberg, 2005, 2006; Randall and Lobel, 2009). In *Elacatinus* gobies there is an association of cleaning behavior with habitat, morphology, and color (Taylor and Hellberg, 2005). The absence of cleaning is associated with sponge-dwelling, while the occurrence of cleaning is related to inhabiting other substrata (Rüber et al., 2003; Taylor and Hellberg, 2005), ideally (but not exclusively) live-coral (Sazima et al., 2000, 2008; Whiteman and Côté, 2002). These alternative ecotypes may also occur intra-specifically (Côté and Soares, 2011), like in the case of the broadstripe cleaning goby *E. prochilos* (Böhlke and Robins, 1968), which in Barbados is known to occupy sponges (sponge-dwellers) and other alternative substrates (mostly live coral) – herein referred to as coral-dwellers (Whiteman and Côté, 2004b). Remarkable changes occur between these two ecotypes of *E. prochilos*; sponge-dwellers live in variable (10 up to 80 individuals), dominance-structured groups of conspecifics, foraging on *Haplosyllis* polychaete worms which parasitize sponges (Colin, 1975; Whiteman and Côté, 2004a). Coral-dwellers, however, are most frequently found in solitary, paired (usually a male-female couple) or in smaller groups of conspecifics, and rely heavily on client-gleaned material as food source (Arnal and Côté, 2000; Whiteman and Côté, 2002). This intraspecific alternative system has been found in other fish species such as *Elacatinus evelynae* from St. Croix, United States Virgin Islands (White et al., 2007) and *Elacatinus figaro* from Brazil (Rocha et al., 2000).

Here, we compare the bacterial communities from the skin and gut of the Barbadian *E. prochilos* alternative ecotypes (sponge- vs. coral-dwelling) sampled in two different localities (biological replicates), to test the hypothesis that different habitat use and behavior will lead to different bacterial profiles in the two ecotypes. Specifically, we hypothesize that the microbiome of cleaners will be enriched by potential pathogens due to frequent contact with diseased clients. To accomplish this aim we will couple high-throughput sequencing of the bacterial 16S rRNA gene V4 region with amplicon sequence variant analysis.

## Materials and Methods

### Sample Collection and DNA Extraction

Twenty-three *E. prochilos* specimens were collected in two reefs located on the west coast of Barbados (13 km apart): Speightstown (sponge-dwellers *N* = 6; coral-dwellers *N* = 6) (13°15′31.8^′′^N 59°38′42.6^′′^W) and Batts Rock (sponge-dwellers *N* = 6; coral-dwellers *N* = 5) (13°08′12.6^′′^N 59°38′16.2^′′^W). Sponge-dwellers formed groups of 5 up to 80 individuals and were associated to giant barrel sponges (*Xestospongia muta*) in the patch reef zone (6–10 m depth). In contrast, coral-dwellers were either solitary or in pairs, mainly associated to live coral, but also other substrates like coralline algae and dead coral from the spurs and grooves zone (3–8 m deep). Sponge-dwellers and coral-dwellers were usually found from 50 to 100 m apart. Fish were captured by SCUBA using individual hand nets or plastic bags and transported to the lab inside sealed plastic bags. In the lab, fish were carefully removed from the bags, and without further manipulation, had their skin swiped with cotton swabs at least two times on each body side. Gloves were used during the procedure. Fish were then sacrificed with an overdose of clove oil mixture. Specimens and cotton swabs were then immediately frozen and kept at -20°C until further analysis. Three weeks later, fish were dissected with sterile material and the whole gut was taken. DNA from 23 skin to 23 guts was extracted using the PowerSoil DNA Isolation Kit (QIAGEN, Netherlands), following the manufacturer’s protocol. DNA concentration and quality was measured in a NanoDrop^TM^ 2000 Spectrophotometer (Thermo Fisher Scientific, United States). Each DNA sample was PCR amplified for the V4 hypervariable region of the 16S rRNA gene (∼250 bp) using the primers F515/R806 developed by Caporaso et al. (2011). This gene region has been widely used to characterize the bacterial communities from vertebrates (Earth Microbiome Project, Gilbert et al., 2014), including fish (Llewellyn et al., 2015; Carlson et al., 2017; Nielsen et al., 2017; Chiarello et al., 2018). Amplicon libraries were prepared using the Dual-Index Sequencing Strategy in Kozich et al. (2013) and sequenced in a single run of the Illumina MiSeq sequencing platform at the Center for Microbial Systems of the University of Michigan Medical School (United States).

### Data and Statistical Analyses

Raw FASTQ files were analyzed using the Quantitative Insights Into Microbial Ecology 2 (QIIME2; release 2018.4) platform. Clean sequences were aligned against the Silva (132) reference database (Quast et al., 2012) with DADA2 pipeline (Callahan et al., 2016). Samples were rarefied to the minimum read count and two feature tables containing amplicon sequence variants (ASVs) from the skin and gut were constructed. The core microbiome was assessed for the skin and gut considering ASVs present in 100% of the samples from each tissue. For the most abundant ASVs in each tissue (>1% of representative sequences), a heatmap was created using the -p-normalize option in QIIME2, which normalizes the feature table by adding a pseudocount of 1 and uses the log10 frequency for the phylum and genus levels.

Bacterial taxonomic alpha-diversity (intra-sample) was calculated using Shannon, Fisher, Faith’s phylogenetic diversity (PD), Evenness, and Simpson indices as implemented in the R package phyloseq (McMurdie and Holmes, 2013). Species beta-diversity (inter-sample) was estimated using Bray–Curtis and phylogenetic Unifrac (unweighted and weighted) distances. Dissimilarity between samples was assessed by principal coordinates analysis (PCoA).

Differences in alpha-diversity across habitat and locality were analyzed by performing a linear model analysis and model effects were evaluated by using 1,000 residual randomizations in a permutation procedure using the R package RRPP (Collyer et al., 2015). Beta-diversity differences across locality and habitat were assessed using permutational analysis of variance (1,000 permutations), as implemented in the *adonis* function of the *vegan* R package. Differences in community composition between ecotypes were tested using linear regression models for the most abundant taxa (with >1% representative sequences).

## Results

### Taxonomic Composition and Core Bacterial Communities in *E. prochilos*

A total of 954,109 raw reads were generated (537,084 for the skin and 417,025 for the gut), with a minimum of 8,991 reads per sample and a maximum of 39,640. These sequences corresponded to 1,155 unique ASVs, from which 662 and 579 were found in the skin and the gut of *E. prochilos*, respectively.

**FIGURE 1:**
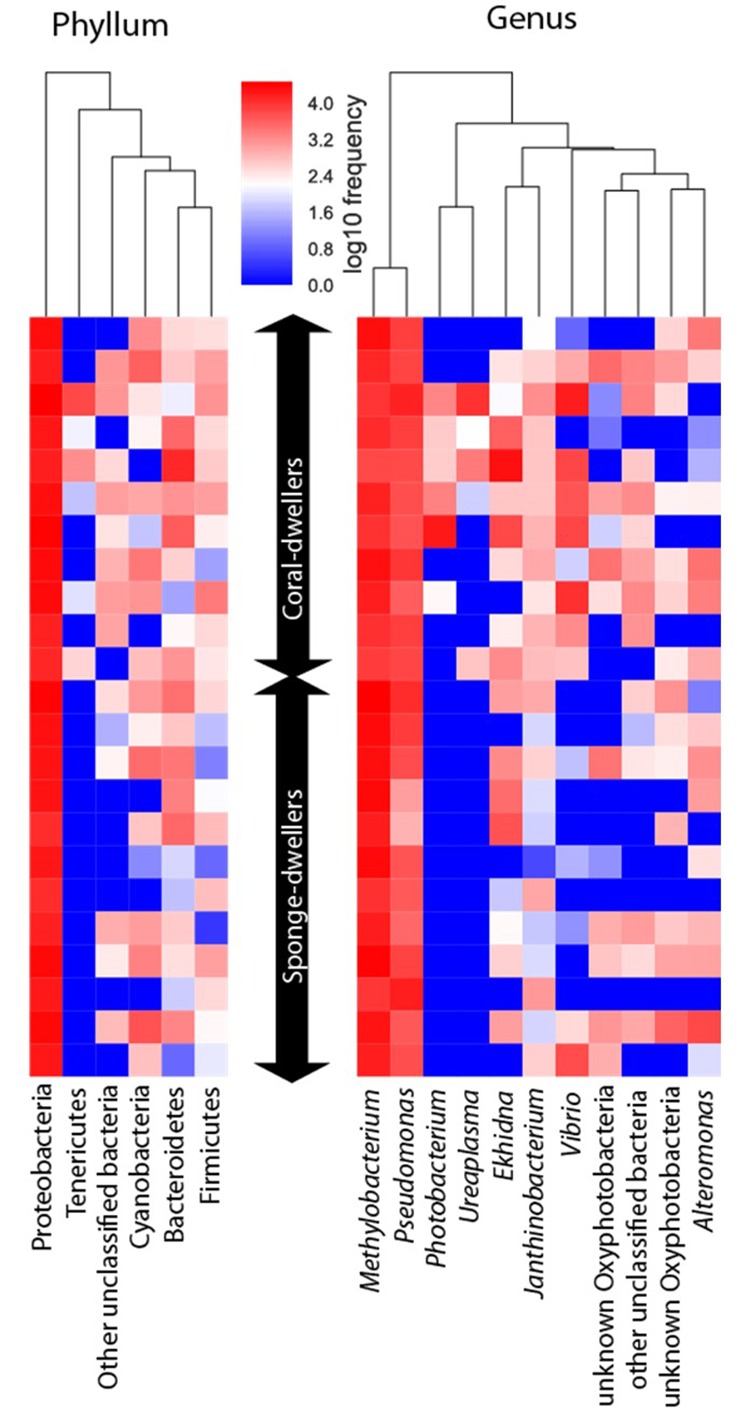
Heatmap depicting absolute abundance of ASVs identified at the phylum and genus levels for the skin microbiome of the two ecotypes.

Twenty-two of the 25 bacterial phyla were detected in the skin, but only five were represented by more than 1% of sequences (Figure 1 and Supplementary Table S1). Members of Proteobacteria (80% of the sequences), Bacteroidetes (7.1%), and Firmicutes (2.2%) occurred in all individuals (Supplementary Table S1), thus forming the core bacterial communities of the skin. Members of Tenericutes (1.6% of the sequences) only occurred in coral-dwellers (but only in 6 out of 11 individuals) (Figure 1 and Supplementary Table S1). Eleven families dominated the core bacterial communities of the skin, with Pseudomonadaceae (ca. 18.5% of the sequences) and Beijerinckiaceae (ca. 47.2%) being the most abundant (Supplementary Table S1). At the genus level, 10 identified genera were represented by >1% of the sequences, with *Methylobacterium* (41.8% of the sequences), *Pseudomonas* (18.5%), and *Janthinobacterium* (1.5%) comprising the core microbiome (Figure 1). It is worth noticing that potential pathogens from the genera *Photobacterium* and *Vibrio*, were more prevalent in coral-dwellers, with the first genus only occurring in this ecotype (Figure 1 and Supplementary Table S1).

**FIGURE 2:**
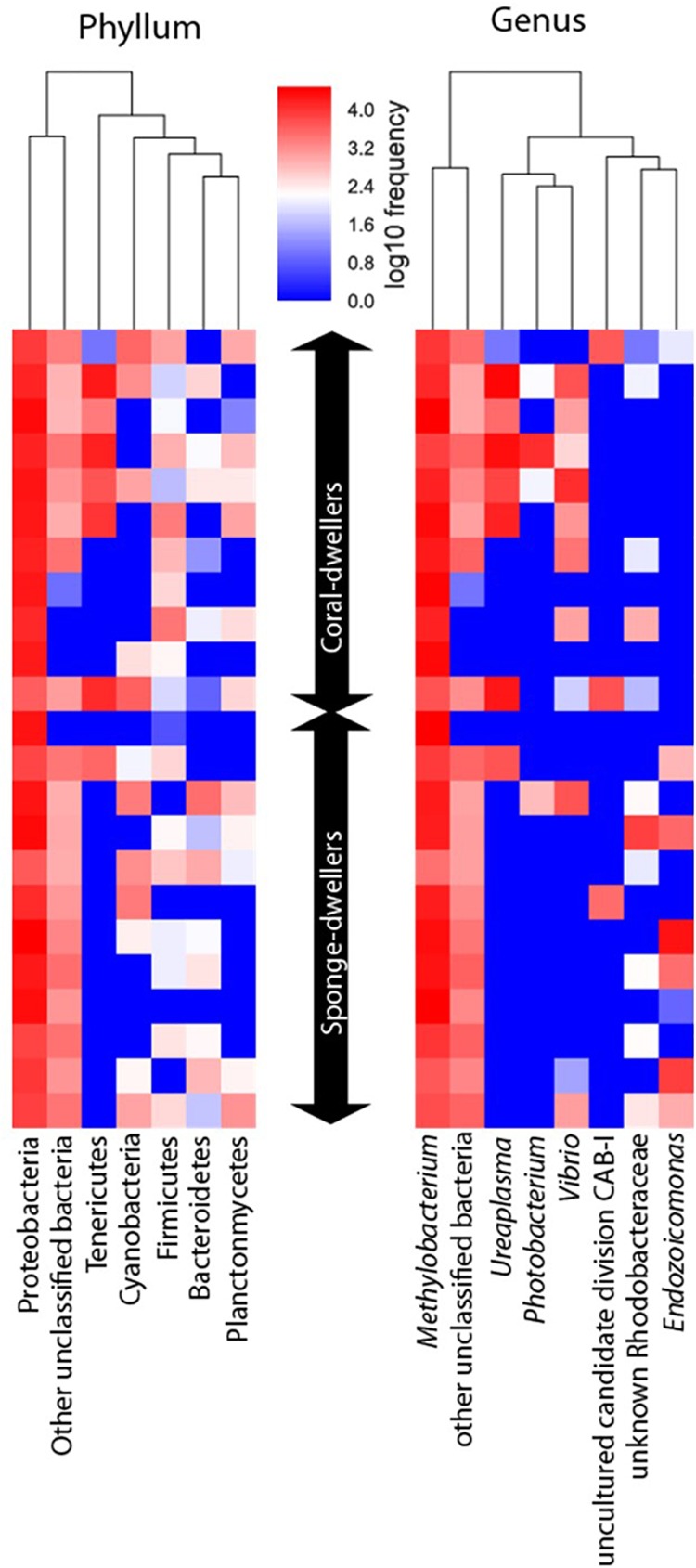
Heatmap depicting absolute abundance of ASVs identified at the phylum and genus levels for the gut microbiome of the two ecotypes.

Nineteen phyla were detected in the gut, but only six were represented by more than 1% of the sequences (Figure 2 and Supplementary Table S2). Only ASVs belonging to Proteobacteria (68% of the sequences) were found in all individuals sampled (Figure 2 and Supplementary Table S2). The core bacterial communities present in the gut were only composed by Beijerinckiaceae, which accounted for 50% of the sequences (Figure 2 and Supplementary Table S2). Seven genera were considered abundant in the gut (Figure 2 and Supplementary Table S2) and from these only *Methylobacterium* (Beijerinckiaceae) (ca. 50% of the sequences) was present in all individuals. As in the skin, potential pathogens from *Photobacterium* and *Vibrio* had higher prevalence in the coral-dwellers (Figure 2).

### Bacterial Diversity Analyses

**FIGURE 3:**
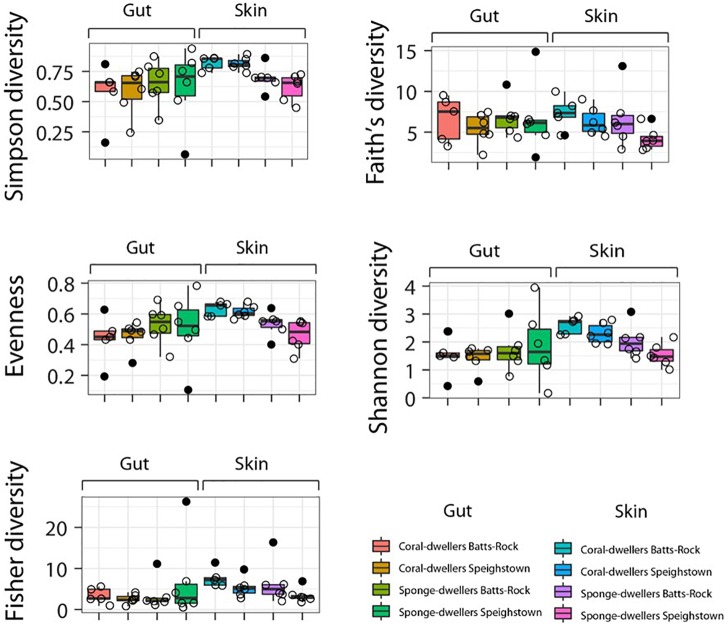
Box-plots depicting mean values and standard deviations of alpha diversity indices for the skin and gut microbiome per locality and ecotype.

**FIGURE 4:**
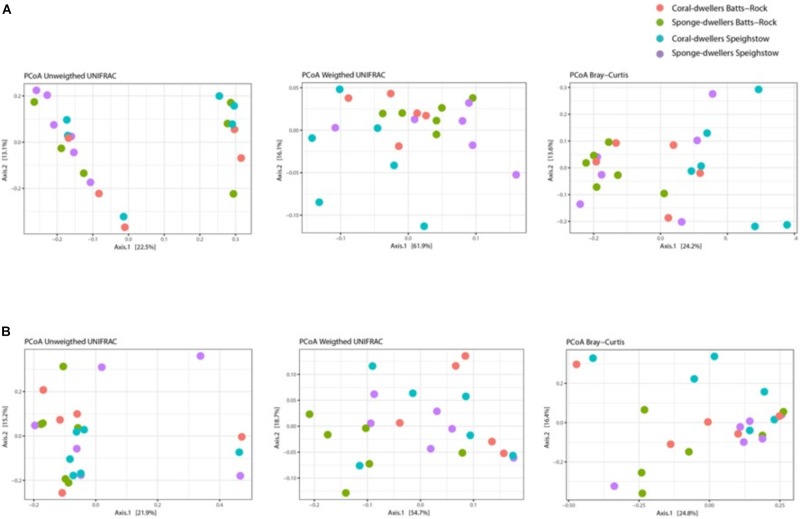
**(A)** PCoA plots depicting weighted and unweighted Unifrac distances and Bray-Curtis distances for the skin microbiome; and **(B)** PCoA plots depicting weighted and unweighted Unifrac distances and Bray-Curtis distances for the gut microbiome.

Skin microbiome alpha-diversity was significantly different between ecotypes, with coral-dwellers showing consistently higher alpha-diversity indices as assessed by Shannon (*F* = 13.786, *p* = 0.002), Simpson (*F* = 20.162, *p* = 0.001), and Evenness (*F* = 17.807, *p* = 0.001) indices (see Figure 3 and Supplementary Tables S3, S4). Locality or the interaction term locality^∗^ecotype had no effect on microbial alpha diversity. Significant differences in beta-diversity were also found between ecotypes using phylogenetic Unifrac weighted (*R*^2^ = 0.224 and *p* = 0.005) and Bray–Curtis (*R*^2^ = 0.098 and *p* = 0.007) distances (Supplementary Table S3 and Figure 4A). Similarly, sampling localities also showed significant differences in beta-diversity for the Bray–Curtis distance (*R*^2^ = 0.087, *p* = 0.018) (Supplementary Table S3 and Figure 4A).

Analysis of mean taxa proportions showed significant differences in the abundance of Vibrionaceae (F-statistics = 6.057, *p* = 0.023) and Beijerinckiaceae (F-statistics = 19.34, *p* < 0.0001) between ecotypes. Within these two families, the abundance of *Vibrio* (Vibrionaceae, F-statistics = 5.443, *p* = 0.030) and *Methylobacterium* (Beijerinckiaceae, F-statistics = 9.32, *p* < 0.0001) varied also significantly between ecotypes (Supplementary Table S1).

For the gut microbiome, locality, ecotype, and the interaction between the two factors had no effect in any of the alpha diversity indices (see Figure 3 and Supplementary Tables S3, S4). However, the analysis of beta-diversity revealed significant differences between ecotypes (*R*^2^ = 0.125, *p* = 0.038) and ecotype^∗^locality for one of the three distances (phylogenetic Unifrac weighted) tested (*R*^2^ = 0.116; *p* = 0.037) (Supplementary Table S3, and Figure 4B). Analysis of mean taxa proportions showed significant differences between ecotypes for *Ureaplasma* (Mycoplasmataceae, Tenericutes) (F-statistics = 6.105; *p* = 0.022), which was almost exclusive of the coral-dwelling ecotype (Supplementary Table S1).

## Discussion

The present study describes the core bacterial communities of the skin and gut of *E. prochilo*s and the differences between two alternative ecotypes (cleaners vs. non-cleaners) in Barbados. Overall, the skin microbiome of coral-dweller obligate cleaners showed consistently greater intra-sample diversity and harbored a significantly higher prevalence of potential fish pathogens confirming our initial hypotheses.

### Taxonomic Composition and Core Bacterial Communities in *E. prochilos*

The skin core microbiome of *E. prochilos* was more diverse than that of the gut, with the former comprised of Proteobacteria, Bacteroidetes, and Firmicutes, whereas the latter only encompassed Proteobacteria of mainly the *Methylobacterium* genus (Beijerinckiaceae). The bacterial profiles reported here for the skin and gut microbiomes of *E. prochilos* are similar to those previously published for other teleosts (e.g., McDonald et al., 2012; Larsen et al., 2014; Lokesh and Kiron, 2016; Carda-Diéguez et al., 2017; Rosado et al., 2019), including other coral reef fish (Chiarello et al., 2018).

### Differences in Bacterial Diversity Between *E. prochilos* Ecotypes

We found significant intraspecific differences in the bacterial diversity of the skin between the two *E. prochilos* ecotypes. The skin microbiome of coral-dwellers had higher alpha diversity (intra-sample) when compared to sponge-dwellers, although observed differences were not always statistically significant. These differences most likely reflect ecotype-specific differences in habitat use, but also behavior, since coral-dwellers depend almost exclusively on client-derived ectoparasites, mucus and tissue for food, coming into frequent contact with other fish species (e.g., Côté and Soares, 2011). Sponge-dwellers, however, by preying on other items, limit their contact with heterospecifics (Whiteman and Côté, 2002). We hence hypothesize that bacterial diversity in the skin of *E. prochilos* can increase by horizontal transfer of bacteria from frequent contact with fish clients. Microbial exchanges via social contact have been reported in several organisms such as chimpanzees (Moeller et al., 2016), ants (Ivens et al., 2018), baboons (Tung et al., 2015), bumblebees (Koch and Schmid-Hempel, 2011), and humans (Kort et al., 2014). In fish, the impact of social transmission on microbiome composition is still unclear, however, there is empirical evidence suggesting that co-housing could have a diluting effect on microbiome differences driven by host genotype (Burns et al., 2016). Importantly, bacterial pathogens have been reported to be transmitted to cleaning fish through contact with diseased clients (Treasurer and Laidler, 1994).

Compared with sponge-dwellers, the skin microbiome of coral-dweller cleaners was significantly enriched with Vibrionaceae (more than 6-fold), a bacterial family known to encompass several fish pathogens (see for example Austin, 2011 for a review). Within this family, ASVs from *Vibrio* and *Photobacterium*, had higher prevalence in the cleaner ecotype. These two genera are known to harbor numerous pathogens able to infect fish worldwide, including tropical species (Landsberg, 1995; Panek, 2005; Gomez-Gil et al., 2007). Indeed, the abundance of *Vibrio* was significantly higher in the skin of coral-dwellers. This supports our initial hypothesis that pathogen transmission may occur from diseased clients.

Taxon differences found in the gut were more subtle. Among the most abundant taxa in the gut, only one ASV belonging to *Ureaplasma* varied significantly between ecotypes. Nonetheless, the interaction between sampling locality and ecotype had an effect on beta-diversity (Weighted Unifrac). This is somewhat surprising since several studies have shown that diet has a significant effect on the gut microbiome composition of fish (see the reviews by Tarnecki et al., 2017; Egerton et al., 2018).

Despite the important ecological role cleaner fish play in marine ecosystems, results from previous studies have provided strong evidence that adopting a non-cleaning lifestyle has some evolutionary advantages, and that sponge-dwelling may be a conservative strategy; for instance, adult *E. prochilos* in Barbados, have been mostly found in sponges regardless of coral habitat availability (Whiteman and Côté, 2004b). This suggests a preference for this habitat or, at least, the existence of potential constraints to adopt a cleaner lifestyle. Importantly, White et al. (2007) found that, overall, immature sponge-dwelling *E. evelynae* gobies grew faster than immature coral-dwellers, and the latter seemed to disappear at higher rates than non-cleaning sponge dwellers. The underlying causes for these disappearances were then suggested to be a result of emigration or were related to higher mortality rates due to predation by clients, which led to the hypothesis that being a cleaner is a riskier and less reliable mode of life, depending heavily on the quality and abundance of clients and more vulnerable to predation (White et al., 2007). Moreover, parasite transmission from diseased clients may also cause this apparent higher mortality, thus representing another negative consequence of adopting a cleaning behavior (Grutter, 2002; Jones et al., 2004). The results from the present study suggest that a higher load of bacterial pathogens may be acquired through cleaning engagement and may help explain the patterns found by White et al. (2007), although more research and data are needed to further confirm this hypothesis.

## Conclusion

The results from this study showed that the bacterial communities of the skin of the two alternative ecotypes of *E. prochilos* can be distinguished using 16S rRNA gene sequences, even amongst fish captured only 10 s of meters apart. Furthermore, the skin microbiome of coral-dwelling gobies (cleaners) harbors higher bacterial diversity, including a significantly higher proportion of potential fish pathogens (e.g., *Vibrio* and *Photobacterium*). We propose that habitat use, diet and social engagement, due to frequent physical contact with potential diseased clients, could lead to significant differences in the diversity and abundance of pathogenic bacteria between cleaner and non-cleaner ecotypes of *E. prochilos*.

## Ethics Statement

This study was carried out in accordance with the recommendations of the Coastal Zone Management Unit (CZMU) in Barbados. The protocol was approved by the Minister of Environment on behalf of the CZUM (permit reference number: CZ01/9/9).

## Author Contributions

RX, MS, and RM conceived the work. RM collected the fish. RX and JS conducted the laboratory work. RX, MP-L, and DR conducted data analysis. All authors contributed to writing the manuscript.

## Conflict of Interest Statement

The authors declare that the research was conducted in the absence of any commercial or financial relationships that could be construed as a potential conflict of interest.
