# Supplementary material for: A Risky Business? Habitat and Social Behavior Impact Skin and Gut Microbiomes in Caribbean Cleaning Gobies
Source: Front Microbiol. 2019 Apr 9;10:716. doi: 10.3389/fmicb.2019.00716 (PMC6467100; doi:10.3389/fmicb.2019.00716)
Supplement: Supplementary file 1 [file Table_1.DOCX]

Table S1: Most abundant ASVs, represented by >1% of the total sequences found for the skin of *E. prochilos*, respective abundances (in percentage), and F-statistics for the comparison between relative abundance of each ASV in each ecotypes. Taxa forming the core microbiome (100% of individuals) and significant differences are depicted in bold.

|  | **TAXA** | **Abundance (%)** | **F-statitics (p-value)** |
| --- | --- | --- | --- |
| **PHYLLUM** | **Bacteroidetes** | 7.1 | 0.552 (0.466) |
|  | **Firmicutes** | 2.2 | 1.93 5(0.179) |
|  | **Proteobacteria** | 80 | 3.621 (0.071) |
|  | Tenericutes | 1.6 | 2.63 (0.120) |
|  | Cyanobacteria | 4.8 | 0.013 (0.911) |
| **FAMILY** | Cyclobacteriaceae | 6 | 0.567 (0.460) |
|  | uncultured diatom | 1,5 | 1.323 (0.263) |
|  | unknown Oxyphotobacteria | 1.8 | 2.074 (0.165) |
|  | ubknow Rhodobacteraceae | 2.7 | 0.104 (0.751) |
|  | Alteromonadaceae | 2.7 | 0.0127 (0.911) |
|  | **Burkholderiaceae** | 1.6 | 0.454 (0.508) |
|  | Moraxellaceae | 1 | 1.428(0.245) |
|  | **Pseudomonadaceae** | 18.4 | 0.021 (0.886) |
|  | Vibrionaceae | 9.1 | **6.057 (0.023)** |
|  | Mycoplasmataceae | 1.6 | 2.554 (0.125) |
|  | **Beijerinckiaceae** | 47.2 | **19.34 (0.000)** |
| **GENUS** | ***Methylobacterium*** | 41.8 | **19.32 (0.000)** |
|  | ***Janthinobacterium*** | 1.5 | 0.333 (0.570) |
|  | ***Pseudomonas*** | 18.4 | 0.021 (0.886) |
|  | *Ekhidna* | 5.6 | 0.766 (0.391) |
|  | unknown Oxyphotobacteria | 1.5 | 1.323 (0.263) |
|  | unknown Oxyphotobacteria | 1.8 | 0.1038 (0.751) |
|  | *Alteromonas* | 2.6 | 0.025 (0.876) |
|  | *Photobacterium* | 2.6 | 2.072 (0.165) |
|  | *Vibrio* | 6.5 | **5.443 (0.030)** |
|  | *Ureaplasma* | 1.6 | 2.554 (0.125) |
