# Supplementary material for: A Risky Business? Habitat and Social Behavior Impact Skin and Gut Microbiomes in Caribbean Cleaning Gobies
Source: Front Microbiol. 2019 Apr 9;10:716. doi: 10.3389/fmicb.2019.00716 (PMC6467100; doi:10.3389/fmicb.2019.00716)
Supplement: Supplementary file 2 [file Table_2.DOCX]

Table S2: Most abundant ASVs, represented by >1% of the total sequences found for the gut of *E. prochilos*, respective abundances (in percentage), and F-statistics for the comparison between relative abundance of each ASV in each ecotypes. Taxa forming the core microbiome (100% of individuals) and significant differences are depicted in bold.

|  | **TAXA** | **Abundance(%)** | **F-statistics (p-value)** |
| --- | --- | --- | --- |
| **PHYLLUM** | **Proteobacteria** | 68 | 0.943 (0.34) |
|  | Tenericute | 13,2 | **6.105 (0.022)** |
|  | Planctomycetes | 1,3 | 0.000 (0.98) |
|  | Firmicutes | 2,2 | 2.715 (0.11) |
|  | Cyanobacteria | 3,8 | 0.016 (0.90) |
|  | Bacteroidetes | 1,3 | 3.421 ( 0.078) |
| **FAMILY** | uncultured candidate division CAB-I bacterium | 2 | 0.669 (0.423) |
|  | **Beijerinckiaceae** | 50 | 0.001 (0.971) |
|  | Rhodobacteraceae | 2 | 0.417 (0.525) |
|  | Endozoicomonadaceae | 5,2 | 3.824 (0.063) |
|  | Moraxellaceae | 1,1 | 0.222 (0.642) |
|  | Vibrionaceae | 6,2 | 3.762 (0.066) |
|  | Mycoplasmataceae | 13,2 | **6.105 (0.022)** |
| **GENUS** | uncultured candidate division CAB-I bacterium | 2 | 0.669 (0.4226) |
|  | ***Methylobacterium*** | 50 | 0.004 (0.952) |
|  | unknown Rhodobacteraceae | 1,5 | 0.862 (0.363) |
|  | *Endozoicomonas* | 5,2 | 3.824 (0.064) |
|  | *Photobacterium* | 1,7 | 1.017 (0.324) |
|  | *Vibrio* | 4,4 | 2.309 (0.144) |
|  | *Ureaplasma* | 13,2 | **6.105 (0.022)** |
