# Supplementary material for: A Risky Business? Habitat and Social Behavior Impact Skin and Gut Microbiomes in Caribbean Cleaning Gobies
Source: Front Microbiol. 2019 Apr 9;10:716. doi: 10.3389/fmicb.2019.00716 (PMC6467100; doi:10.3389/fmicb.2019.00716)
Supplement: Supplementary file 3 [file Table_3.docx]

Table S3: Summary of results obtained from the linear model (lm.RRPP) using alpha-diversity indexes as dependent variable (F-statistics and respective p-values), and from permutational multivariate analysis of variance (adonis function) for beta-diversity estimates (R^2^ and respective p-values), for skin and gut microbiome. Significant associations are depicted in bold.

|  | **Alpha-diversity indexes** | **Locality** | **Habitat (ecotype)** | **Locality***  **Habitat** |
| --- | --- | --- | --- | --- |
| **Skin** | **PD** | 3.279 (0.066) | 2.352 (0.122) | 0.493 (0.456) |
|  | **Evenness** | 1.929 (0.169) | **17.807 (0.001)** | 0.791 (0.230) |
|  | **Fisher** | 3.479 (0.060) | 1.429 (0.220) | 0.055 (0.814) |
|  | **Shannon** | 4.048 (0.051) | **13.786 (0.002)** | 0.370 (0.391) |
|  | **Simpson** | 0.911 (0.337) | **20.162 (0.001)** | 0.974 (0.177) |
|  | **Uni Un** | 0.053  (p= 0.231) | 0.061 (p=0.126) | 0.062 (p=0.093) |
|  | **Uni Weigh** | 0.060  (p= 0.239) | **0.224 (p=0.005)** | 0.047 (p=0.243) |
|  | **Bray C** | **0.087**  **(p= 0018)** | **0.098 (p=0.007)** | 0.044 (p=0.34) |
| **Gut** | **PD** | 0.289 (0.624) | 0.369 (0.570) | 0.195 (0.679) |
|  | **ACE** | 0.010 (0.911) | 1.040 (0.307) | 0.092 (0.754) |
|  | **Fisher** | 0.276 (0.756) | 1.023 (0.424) | 0.765 (0.455) |
|  | **Shannon** | 0.018 (0.886) | 0.889 (0.367) | 0.097 (0.754) |
|  | **Simpson** | 0.013 (0.917) | 0.325 (0.559) | 0.031 (0.856) |
|  | **Uni Un** | 0.047 (p=0.377) | 0.047 (p=0.366) | 0.042 (p=0.495) |
|  | **Uni Weigh** | 0.044 (0.389) | **0.125 (p=0.038)** | **0.116 (p=0.037)** |
|  | **Bray C** | 0.053 (p=0.244) | 0.068 (p=0.086) | 0.034 (p=0.693) |
