# Supplementary material for: A Risky Business? Habitat and Social Behavior Impact Skin and Gut Microbiomes in Caribbean Cleaning Gobies
Source: Front Microbiol. 2019 Apr 9;10:716. doi: 10.3389/fmicb.2019.00716 (PMC6467100; doi:10.3389/fmicb.2019.00716)
Supplement: Supplementary file 4 [file Table_4.DOCX]

|  | Locality | Habitat/ecotype | Evenness (CI) | Fisher (CI) | PD (CI) | Shannon (CI) | Simpson (CI) |
| --- | --- | --- | --- | --- | --- | --- | --- |
| **Skin** | Batts Rock | coral-dwellers | 0.634 (0.599; 0.666) | 7.63 (6.23; 9.96) | 7.41 (5.69; 8.76) | 2.59 (2.36; 2.81) | 0.818 (0.762; 0.858) |
|  |  | sponge-dwellers | 0.535 (0.465; 0.584) | 6.35 (3.82; 12.5) | 6.64 (4.68; 10.20) | 2.04 (1.69; 2.57) | 0.694 (0.622;0.776) |
|  | Speighstown | coral-dwellers | 0.614 (0.586; 0.649) | 5.38 (4.03; 7.76) | 6.31 (5.19; 7.73) | 2.31 (2.06; 2.59) | 0.813 (0.776; 0.854) |
|  |  | sponge-dwellers | 0.462 (0.381; 0.524) | 3.48 (2.59; 5.36) | 4.18 (3.41;5.51) | 1.54(1.26; 1.87) | 0.618 (0.520; 0.687) |
| **Gut** | Batts Rock | coral-dwellers | 0.439 (0.293; 0.533) | 3.35 (1.91; 4.76) | 6.63 (4.20; 8.55) | 1.47 (0.848; 1.90) | 0.575 (0.260; 0.705) |
|  |  | sponge-dwellers | 0.529 (0.421; 0.607) | 3.56 (1.87; 8.25) | 6.81 (5.50; 8.90) | 1.70 (1.24; 2.39) | 0.651 (0.493; 0.767) |
|  | Speighstown | coral-dwellers | 0.456 (0.350; 0.505) | 2.55 (1.72; 3.37) | 5.42 (3.70; 6.54) | 1.42 (0.903; 1.64) | 0.585 (0.387; 0.690) |
|  |  | sponge-dwellers | 0.505 (0.293; 0.639) | 6.78 (2.07; 18.40) | 6.71 (4.31; 10.60) | 1.87 (0.998; 2.96) | 0.626 (0.290; 0.792) |
|  |  |  |  |  |  |  |  |
|  |  |  |  |  |  |  |  |

Table S4: Mean values for alpha-diversity indices with 95% confidence levels
